# The use of dietary supplements for mental health among the Saudi population: A cross-sectional survey

**DOI:** 10.1016/j.jsps.2022.03.017

**Published:** 2022-03-29

**Authors:** Deemah Alateeq, Maha A. Alsubaie, Faridah A. Alsafi, Sultanah Hisham Alsulaiman, Ghazwa B. Korayem

**Affiliations:** a Clinical Sciences Department, College of Medicine, Princess Nourah bint Abdulrahman University, Riyadh, Saudi Arabia; b Pharmaceutical Care Services, King Abdullah bin Abdulaziz University Hospital, Riyadh, Saudi Arabia; c Health Sciences Research Center (HSRC), Princess Nourah bint Abdulrahman University, King Khalid International Airport, Riyadh, Saudi Arabia; d Department of Pharmacy Practice, College of Pharmacy, Princess Nourah bint Abdulrahman University, Riyadh, Saudi Arabia

**Keywords:** Dietary supplements, Anxiety, Depression, Sleep, DS, dietary supplements, OR, odds ratio, PHQ-9, patient health questionnaire, GAD-7, generalized anxiety disorder questionnaire, ISI, insomnia severity index, SA, Saudi Arabia, CI, confidence level, PNU, Princess Nourah bint Abdulrahman University, IRB, institutional review board, SD, standard deviation

## Abstract

**Introduction:**

Despite limited evidence about the efficacy and safety of dietary supplements (DSs) for improving mental health, people with or without mental disorders often tend to use them, especially during the ongoing COVID-19 pandemic. Previous studies focused on DS use for maintaining or improving overall health; Therefore, this study aimed to assess the prevalence of DSs for mental health among the SA population and to determine the factors that affect their use.

**Methods:**

This cross-sectional study was based on an online survey of Saudi Arabian participants between July and August 2021 with an anonymous, self-completed questionnaire distributed using convenience sampling. The questionnaire included queries related to demographic information, DS use assessment, and mental health evaluation using the Patient Health Questionnaire (PHQ-9), the Generalized Anxiety Disorder 7-item (GAD-7), questionnaire, and the Insomnia Severity Index (ISI).

**Results:**

In total, 443 participants from various regions of Saudi Arabia completed the questionnaire. The prevalence of DS use in the Saudi population was 44%. Vitamin D (28%) and melatonin (20%) were the most commonly reported DSs used for mental health. The odds of DS use were three times higher in responders with previous mental health diagnoses (OR: 2.972; 95% CI: 1.602–5.515). Furthermore, the chances of using DSs almost doubled in patients with sub-threshold and moderate to severe insomnia (OR: 1.930; 95% CI: 1.191–3.126 and OR: 2.485; 95% CI: 1.247–4.954, respectively).

**Conclusion:**

Responders diagnosed by a specialist with psychiatric disorders or current insomnia had a higher chance of using DSs. Thus, healthcare providers must provide evidence-based information regarding DSs for mental health improvement and encourage the public to consult healthcare professionals before self-medicating for mental health problems.

## 1 Introduction

Mental health is an essential element of public healthcare, and there has been a rapid global increase in related cases recently (Saxena et al., 2007) (Trautmann et al., 2016). In 2010, mental and substance-use disorders were the leading causes of years living with disability (Whiteford et al., 2013). Currently, the estimated prevalence of mental health disorders in Saudi Arabia (SA) is approximately 34.2%, with a lifetime morbidity risk of 38% (Altwaijri et al., 2020). Among those with a lifetime mental disorder, 23.2% cases had anxiety, 6% had depression, and 4% had a substance-related disorder (Altwaijri et al., 2020), while the prevalence of insomnia in SA is reported to be 77.7% (Ahmed et al., 2017). These numbers highlight the need for effective treatment of affected individuals (Ahmed et al., 2017, Altwaijri et al., 2020). Recently, the Saudi National Mental Health Survey reported that although the majority of the general Saudi population had free access to mental health services, only 13.7% of those with psychiatric disorders had actually received treatment (Altwaijri et al., 2020). The main barrier to seeking mental health treatment reported was patient self-help preference (Alangari et al., 2020). Therefore, patients with psychiatric disorders may seek alternative treatment options such as dietary supplements (DSs).

DS use has increased appreciably in the last 30 years (Bailey et al., 2013). The rate of DS use in the United States (U.S.) nearly tripled, from 18.9% to 49%, between 2002 and 2010 (Bailey et al., 2017, Kennedy, 2005). This significant increase in the use of DSs for treating mental health disorders was mainly due to the easy purchase of DSs (Baume et al., 2006). At the same time, there is limited accessibility of pharmacological treatments, especially for low-income patients (Baume et al., 2006, Kennedy, 2005). Furthermore, DSs are usually perceived as a safer alternative to pharmacotherapy, with lower risks of adverse effects and withdrawal (Algaeed et al., 2019). In contrast, prescribed treatment can be problematic for several reasons, including access to healthcare, treatment restrictions (healthcare specialist prescriptions), and other factors related to patient beliefs.

A national household survey conducted in the U.S. between 1997 and 1998 reported that 16.5% of respondents used DSs to help with emotional, mental health, and alcohol or drug abuse related problems (Unützer et al., 2000). Of these responders, 21% met the diagnostic criteria for one or more psychiatric disorders, compared to 12.8% of respondents who did not report DS use (Unützer et al., 2000). Meanwhile, in SA, a recent cross-sectional study conducted in Riyadh reported the prevalence of DS use as 63.2% (Algaeed et al., 2019). However, most previous studies have focused solely on assessing the use of DSs for general health and well-being (Algaeed et al., 2019, Bailey et al., 2013, Kennedy, 2005).

Until now, there is limited evidence for DSs being beneficial in improving symptoms related to mental health disorders (Lakhan and Vieira, 2008). A recent randomized control trial (RCT) found that vitamin D and omega-3 co-supplementation improved depression, anxiety, and sleep quality in women with prediabetes and hypovitaminosis D (Rajabi-Naeeni et al., 2019). Furthermore, a *meta*-analysis of RCTs suggested that DSs such as omega-3 fatty acids and N-acetylcysteine may be beneficial for treating people with mental disorders under certain conditions (Firth et al., 2019). The American Psychological Association (APA) guideline for the treatment of depression also conditionally recommends using St. John’s Wort for adults with depression for whom psychotherapy or pharmacotherapy is ineffective (American Psychological Association, 2019), although the evidence is still insufficient to support its use in patients with mild depression (American Psychological Association, 2019).

During the COVID-19 pandemic, the use of DSs is reported to have increased significantly (Hamulka et al., 2020). This increase led the U.S. Food and Drug Administration (FDA) to issue a warning to companies that were selling DSs by claiming that they could treat depression or other mental health disorders in February 2021 (U.S Food & Drug Administration, 2021). Even though evidence supporting the use of DSs for mental health improvement is limited (Firth et al., 2019), consumers especially tended to use DSs during the ongoing COVID-19 pandemic as people were more susceptible to depression and other mental health disorders (BinDhim et al., 2021). Therefore, it is essential to assess the public's attitude towards using DSs for mental health. This study aimed to assess the prevalence of DSs for mental health among the SA population and to determine the factors that affect their use.

## 2 Material and methods

### 2.1 Study design and population

This cross-sectional survey was conducted across various regions of SA between July and August 2021. The data were collected using an online anonymous self-completed questionnaire in Arabic distributed using convenient non-probability sampling techniques via email and social media channels. The inclusion criteria were as follows: adults aged 18 years or older who spoke Arabic and were residents of SA. Responders who were under 18 years of age and those who did not complete all survey sections were excluded. In 2019, the General Authority for Statistics in SA reported the general population of SA as 34,218,169. The calculated sample size to meet power was 385 (Kingdom of Saudi Arabia General Authority of Statistics, 2021). Using Raosoft (Seattle, WA, USA), it was estimated to be at the 95% confidence level with a 50% response distribution and margin of error of ±5%.

### 2.2 Study design and population

A panel of experts on the research subject reviewed the questionnaire for face validity and agreed that the items were clear, comprehensive, and suitable for the study purpose, with minor modifications being made based on their feedback. The questionnaire was developed in English and then translated into Arabic and included three main sections. The first section contained 13 questions about the responders' demographic information, including age, gender, marital status, educational level, employment, monthly income, nationality, region of residence, smoking status, substance use, including alcohol, medical condition, and previous history of diagnosis and treatment for mental health disorders. The second section comprised several questions on assessing the use of DSs for mental health. The scientific names of 40 DSs used previously and currently for improving mental health were listed, and data was obtained regarding the sources of information regarding these DSs as well as the reasons for using or not using them. The number of questions was subject to change depending on the respondent’s answers, as we applied branching. The third section of the questionnaire was on mental health assessment using validated screening tools. We used validated Arabic versions of the General Anxiety Disorder 7-item (GAD-7) questionnaire, the Patient Health Questionnaire (PHQ-9), and the Insomnia Severity Index (ISI) (AlHadi et al., 2017, Hallit et al., 2019). Knowing that these tools can only assess anxiety and depression on symptomatic basis and not as a definite diagnosis.However, all participants were asked questions related to anxiety, depression, and insomnia symptom severity regardless of their history of previous diagnosis of anxiety, depression, or sleep disorder.

The GAD-7 contains seven questions used to screen for anxiety in the last four weeks, where the total score ranges from 0 to 21, with a possible score of 0–3 points for every question (Spitzer et al., 2006). A score of 0–4 indicates minimal anxiety, a score of 5–9 indicates mild anxiety, a score of 10–14 indicates moderate anxiety, and a score of 15 or above indicates severe anxiety (Spitzer et al., 2006).

• The PHQ-9 questionnaire assesses depression severity and functional health (Kroenke et al., 2001). The tool evaluates nine depression symptoms and asks the participant to rate the frequency of symptoms in the last two weeks, ranging from “not at all” (score = 0) to “nearly every day” (score = 3) (Kroenke et al., 2001). The total score ranges between 0 and 27, where a score of 0–4 indicates minimal or no depression, 5–9 indicates mild depression symptoms, 10–14 indicates moderate depression symptoms, 15–19 indicates moderate severity of depression symptoms, and 20–27 indicates severe depression symptoms (Kroenke et al., 2001).
• The ISI was used to assess the severity of insomnia (Bastien, 2001). It includes seven questions on the nature of sleep problems in the last two weeks. In the first three questions, responders were required to rate the severity of their symptoms as none (0), mild (1), moderate (2), severe (3), or very severe (4). The remaining questions were about satisfaction with sleep pattern, quality of life impairment, distress, and symptoms interfering with daily function. The answers to these questions are scores on a five-point Likert scale ranging from 0 (none) to 4 (very much). The total score ranges from 0 to 28 (Bastien, 2001). A score between 0 and 7 indicates no clinically significant insomnia, 8–14 indicates subthreshold insomnia, 15–21 indicates clinical insomnia of “moderate severity,” and 22–28 indicates “severe” clinical insomnia (Bastien, 2001).

Responders who had used a DS for mental health at any point in their lives (before responding to the survey or even those currently using DS) were categorized as “DS users,” whereas responders who reported not using them in the past or currently were grouped as “DS non-users.” To avoid limits on the responders’ understanding of “mental health” vs. psychiatric disorders, we used the terms “psychiatric disorders” and “sleep disorders” for specificity in the questionnaires.

### 2.3 Ethics

Ethical approval was obtained from the Institutional Review Board of Princess Nourah Abdulrahman University (PNU) and the Ethical Approval Committee (IRB Log number 21-0305) in accordance with the Declaration of Helsinki. Consent was obtained from the patients after they had read the study description and voluntarily agreed to participate. All data were collected and stored anonymously using the Research Electronic Data Capture (ReDCap®) 7.3.6 software, hosted by PNU.

### 2.4 Statistical analysis

Data management and analysis were performed using Statistical Package for Social Sciences (SPSS v. 24, Armonk, NY, USA). Categorical variables are presented as frequencies and percentages, whereas continuous variables are presented as means and standard deviations (SDs). The chi-square test was used to assess the association between demographic characteristics and the use of DSs. Multivariate logistic regression, and backward elimination were used to examine mental health/sleep (independent variables) and DS use (dependent variable). The variable entry *p*-value was set at 0.5 and the elimination *p*-value was set at 0.05. Independent sample t-tests were used to assess whether the mean scores of DS users and non-users were significantly different. Logistic regression results were reported as odds ratios (ORs) with 95% confidence intervals (CIs). The significance level was set at *p* < 0.05.

## 3 Results

A total of 675 respondents filled the questionnaire. However, only 443 patients were included in the study. The remaining 242 responders were excluded for the following reasons: 181 did not complete all sections, 57 were identified as duplicates, and four were less than 18-years-old. Thus, the overall response rate was 71%.

### 3.1 Demographic characteristics

The mean age of respondents was 37.56 years. The study population was predominantly Saudi (94%), and 73% were women from the central region (62%). Overall, 16% of the responders had a previous diagnosis of a psychiatric disorder, of whom 38% had depression, 24% had anxiety, and 19% had sleep disorders. In addition, 68% of patients reported being prescribed psychotropic medication. Detailed demographic characteristics are presented in Table 1.

**Table 1:** Demographic characteristics of survey responders (N = 443).

Characteristics	Overall (N = 443)	DS users (N = 194)	Non DS users (N = 249)	P-value
**Age, mean (SD)**	37.56 (13)	36.18 (11.58)	38.63 (14.38)	0.053

*Sex, n (%)*				0.683
Male	119 (27)	54(27.8)	65(26.1)	
Female	324 (73)	140(72.2)	184(73.9)	

*Marital status, n (%)*				0.429
Single	178 (40)	85 (43.8)	93 (37.3)	
Married	228 (51.5)	95 (49)	133 (53.4)	
Divorced	29 (6.5)	12(6.2)	17 (6.8)	
Widowed	8 (2)	2(1)	6 (2.4)	

*Education level, n (%)*				0.108
High school or below	30 (7)	9 (4.6)	21 (8.4)	
Diploma	26 (6)	9 (4.6)	17 (6.8)	
Bachelors	249 (56)	104 (53.6)	145 (58.2)	
Masters	70 (16)	36 (18.6)	34 (13.7)	
PhD	68 (15)	36(18.6)	32(12.9)	

*Employment, n (%)*				**0.030**
Student	64 (14)	25 (12.9)	39 (15.7)	
Employee	172 (39)	84 (43.3)	88 (35.3)	
Unemployed/ Retired	78 (18)	26 (13.4)	52 (20.9)	
Housewife	54 (12)	18 (9.3)	36 (14.5)	
Freelancer	16 (4)	10 (5.2)	6 (2.4)	
Healthcare provider	59 (13)	31 (16)	28 (11.2)	

*Monthly income, n (%)*				**0.048**
< 5000 SAR	26 (6)	7 (3.6)	19 (7.6)	
5000 to 10,000 SAR	80 (18)	28 (14.4)	52 (20.9)	
10,000 to 15,000 SAR	94 (21)	48(24.7)	46 (18.5)	
> 15,000 SAR	243 (55)	111 (57.2)	132 (53)	

*Nationality, n (%)*				0.465
Saudi	416 (94)	184 (94.8)	232 (93.2)	
Non-Saudi	27 (6)	10 (5.2)	17 (6.8)	

*Region of living, n (%)*				0.445
Northern region	12 (3)	2 (1)	10 (4)	
Eastern region	40 (9)	18 (9.3)	22 (8.8)	
Central region	275 (62)	123(63.4)	152 (61)	
Western region	105 (24)	46 (23.7)	59 (23.7)	
Southern region	11 (2)	5 (2.6)	6 (2.4)	

*Smoking status, n (%)*				0.115
Non-smoker	355 (80)	147 (75.8)	208 (83.5)	
Ex-smoker	19 (4)	11 (5.7)	8 (3.2)	
Smoker	69 (16)	36 (18.6)	33 (13.3)	

*History of substance use, n (%)*				0.082
	19 (4)	12 (6.2)	7 (2.8)	

*Admits to drinking alcohol, n (%)*				0.975
	50 (11)	22 (11.3)	28 (11.2)	
*Positive history of diagnosis of a chronic medical condition(s), n (%)*	118 (27)	58 (29.9)	60 (24.1)	0.171
*Positive history of diagnosis of a mental disorder, n (%)*	72 (16)	50(25.8)	22(8.8)	**0.000**

*Positive history of psychiatric disorders diagnoses by a specialist, n (%)*				0.482
Depression	27 (38)	19 (38)	8 (36.4)	
Anxiety	17 (24)	12 (24)	5 (22.7)	
Post-traumatic stress disorder	1 (1)	–	1 (4.5)	
Panic disorder	7 (10)	4 (8)	3 (13.6)	
Bipolar disorder	2 (3)	2(4)	–	
Psychosis	1 (1)	–	1 (4.5)	
Sleep disorders	14 (19)	11 (22)	3 (13.6)	
Other	3 (4)	2 (4)	1 (4.5)	
*Prescribed psychotropic medication, n (%)*	49 (68)	33 (66)	16 (72.7)	0.573

The differences between DS users and non-users are shown in Table 1. The baseline characteristics between DS users and non-users were balanced in terms of most characteristics, except employment, income, and a history of mental health disorders. Employment status and monthly income were significantly associated with DS use (*p* = 0.030 and 0.048, respectively). A previous mental health disorder diagnosis was also significantly associated with DS use (*p* < 0.001).

### 3.2 Use of DSs

The overall prevalence of DSs in terms of improving mental health and sleep was 44%. However, 170 (87%) responders admitted to using the supplement at the time of survey completion. Among the 40 DSs listed in the survey, vitamin D was the most used supplement (28%), followed by melatonin (20%), and vitamin B12 and omega 3 (18% each). Gingko, St. John’s wort, and Kava Kava were used by < 5% of the participants who admitted using DS. The complete list of commonly used DSs and their frequency of use are presented in Table 2. Most participants did not experience side effects when using DSs (62.4%). Regarding the reason for DS use, 23% of the participants believed that “DSs improve mental health.” In comparison, 14% and 11% used DSs based on advice from a health professional or from a friend, relative, or colleague, respectively. In addition, the most common source participants reported referring to for information related to DSs was the internet (48.3 %), while psychiatrists were further down the list (9.5 %), as presented in Supplementary Table S1. The reported reasons for using or avoiding DS are listed in Table 3. The most commonly reported reasons for avoiding DS use among non-DS users were that they felt that they did not need it (40%), followed by 20% who did not believe it would help with mental health. DS use was reported primarily for sleep disorders (13%), followed by anxiety (10%) and depression (7%).

**Table 2:** Dietary Supplements name, frequency, sources of information, and side effects reported being used by DS users (N = 194).

DS name	N, (%)
Vitamin D	124 (64)
Melatonin	87 (45)
B12	80 (41)
Omega 3/fish oil	78 (40)
Vitamin C	63 (32)
Magnesium	51 (26)
Zinc	50 (25.8)
Vitamin B complex	41 (21)
Chamomile	39 (20)
Folic acid	38 (19.6)
Vitamin E	22 (11.3)
Probiotics	14 (7.2)
Vitamin K	11 (5.7)
Gingko	8 (4.1)
Ashwagandha	8 (4.1)
Acetyl-L-carnitine	6 (3.1)
St. John's wort	5 (2.6)
Lipidated curcumin	5 (2.6)
Rhodiola rosea	4 (2.1)
Kava Kava	1 (0.5)
Others*	17 (8.7)
Do not recall the name	18 (9.3)

*Frequency of DS use*	
Always	42 (21.6)
Often	42 (21.6)
Sometimes	69 (35.6)
Rarely	40 (20.6)
Never	1 (0.5)

*Experienced side effects when using DS*	
Yes	32 (16.5)
No	121 (62.4)

Others (<2%): Tryptophan, N-acetylcysteine, Lion's Mane, Relora® (magnolia and phellodendron bark), lecithin, L-tyrosine, hydroxytryptophan, L-theanine, lemon balm (Melissa officinalis), Feverfew herb, choline, phenylalanine.

**Table 3:** Surveyors reported reasons for using DSs vs. not using DSs.

Users (N = 194)	N (%)	Non-users (N = 249)	N (%)
DS improves mental health	96 (21.7)	I don't need it	178 (40.2)
Advice from a health professional	61 (13.8)	I didn't think of it as a treatment to improve mental health	90 (20.3)
Advice from a friend, relative, or colleague	49(11.1)	My information on its effectiveness is insufficient	68 (15.3)
DSs help prevent psychiatric disorders/sleep disorders	41 (9.3)	No reason	62 (14)
Fearing the side effects of medications	36 (8.1)	Fearing the side effects of dietary supplements	49 (11.6)
Psychotropic/sleep medications require a prescription	19 (4.3)	I don't believe in the effectiveness of DSs for mental health	26 (5.9)
DS stimulates the effectiveness of psychotropic/sleep medications	15 (3.4)	It is not safe	23 (5.2)
Psychotropic medications are addictive	11 (2.5)	High cost of DSs	19 (4.3)
Psychotropic medications make me feel stigmatized	6 (1.4)	A health professional advised me not to take it	6 (4.1)
Psychotropic medications are not effective	5 (1.1)	Other	9 (2)
Psychotropic/sleep medications are expensive	3 (0.7)		
Other	12 (2.7)		

### 3.3 Association between mental disorders and DS use

Data regarding the comparison of DS use between responders with anxiety, depression, and insomnia is shown in Table 4. The mean score for depression symptoms was found to be significantly higher among DS users (mean and standard deviation [SD]: 8.23 [6.18] vs. non-DS users 6.57 [5.89]; t (4 4 1) = −2.881; *p* = 0.004). A significant difference in insomnia symptoms scores was detected between DS users and non-DS users (10.99 [5.05]) vs. 8.75 [4.58]; t (4 4 1) = −4.888; *p* < 0.000). However, the difference in mean scores for anxiety symptoms was not statistically significant (*p* = 0.139).

**Table 4:** Comparing anxiety, depression, and insomnia severity scores among users and non-users of DSs.

	DSs use				
	Users	Non-users	Mean difference	t-value	P-value
	Mean (SD)	Mean (SD)			
GAD-7 score	7.59 (5.29)	6.84 (5.26)	−0.749	−1.484	0.139
PHQ-9 score	8.23 (6.18)	6.57 (5.89)	−1.661	−2.881	**0.004**
ISI score	10.99 (5.05)	8.75 (4.58)	−2.243	−4.888	**0.000**

### 3.4 Predictors of DS use

Following multivariate logistic regression analysis, the unadjusted analysis showed that mild levels of anxiety, mild and moderate levels of depression, sub-threshold insomnia, and moderately severe insomnia were significantly associated with DS use (*p* < 0.05). However, after controlling for potential confounders, the differences in terms of responders with anxiety and depression were not statistically significant. The predictors of DS use were insomnia and a history of mental health disorder diagnosis. According to the ISI scores, participants with sub-threshold insomnia and moderately severe insomnia were almost twice as likely to use DSs compared to those who did not have insomnia (OR: 1.930, 95% CI: 1.191–3.126 and OR: 2.485, 95% CI: 1.247–4.954, respectively). Participants diagnosed previously with mental health disorders were almost three times more likely to use DSs than those who were not (OR: 2.972, 95% CI: 1.602–5.515). Results of the logistic regression analysis for DS use predictors among the participants is shown in Table 5.

**Table 5:** Logistic regression analysis for DS predictors among surveyors.

Parameter	Crude		Multivariate adjusted	
	OR (95% CI)	P-value	OR (95% CI)	P-value
*Employment status*				
Student				
Employee	1.489 (0.830–2.671)	0.182	1.542 (0.823–2.889)	0.176
Unemployed/ Retired	0.780 (0.392–1.552)	0.479	0.793 (0.378–1.663)	0.539
Housewife	0.780 (0.366–1.662)	0.520	0.727 (0.319–1.656)	0.448
Freelancer	2.600 (0.840–8.047)	0.097	2.816 (0.842–9.418)	0.093
Healthcare provider	1.727 (0.844–3.356)	0.135	1.886 (0.862–4.128)	0.112

*Any previous diagnosis of a mental health disorder*				
No				
Yes	3.583 (2.081–6.167)	**0.000**	2.972 (1.602–5.515)	**0.001**

*General Anxiety Disorder-7*				
Lack of anxiety				
Mild anxiety	1.599 (1.002–2.552)	**0.049**	1.067(0.617–1.844)	0.816
Moderate anxiety	1.339 (0.716–2.504)	0.360	0.667(0.311–1.432)	0.299
Severe anxiety	1.291 (0.674–2.471)	0.441	0.637(0.275–1.478)	0.294

*Patient Health Questionnaire-9*				
No depression				
Mild depression	1.677 (1.036–2.715)	**0.035**	1.394 (0.788–2.464)	0.254
Moderate depression	1.969 (1.104–3.514)	**0.022**	1.778 (0.882–3.585)	0.108
Moderately severe depression	2.129 (0.942–4.812)	0.069	1.923 (0.726–5.093)	0.188
Severe depression	1.234 (0.480–3.169)	0.662	1.003 (0.312–3.224)	0.996

*Insomnia Severity Index*				
No clinical insomnia				
Sub-threshold insomnia	2.067 (1.318–3.240)	**0.002**	1.930 (1.191–3.126)	**0.008**
Clinical insomnia (moderate severity)	2.571 (1.397–4.730)	**0.002**	2.485 (1.247–4.954)	**0.010**
Severe insomnia	6.957 (0.719–67.363)	0.094	9.616 (0.882–104.886	0.063

## 4 Discussion

The prevalence of DS use in the Saudi population reported in this study (44 %) is lower than that reported in a previous study conducted in SA, which showed that 63.2% of the responders consumed DSs (Algaeed et al., 2019). This percentage is also lower than the previous rate of DS use in the U.S. reported by the National Health and Nutrition Examination Survey (NHANES), which showed that 57.6% of U.S. adults used DSs between 2017 and 2018 (Mishra Suruchi et al., 2021). Several cross-sectional studies have been conducted to assess DS use among students in SA and the U.S. (Alfawaz et al., 2017, Axon et al., 2017, Chandika et al., 2017). In SA, 76.6% of female college students, 55.75% of medical science students, and 53% of health sciences students used DSs (Alfawaz et al., 2017, Alowais and Selim, 2019, Chandika et al., 2017). In contrast, 52% of pharmacy students used DSs in the U.S. (Axon et al., 2017). The high rate of DS use in this study may be driven by the convenience of purchasing these supplements, especially online. In addition, responders may have been influenced by marketing advertisements and information shared on the internet, as it was reported to be the main source of DS information in this study.

In this study, vitamin D was the most common DS used (28%), followed by melatonin (20%) and vitamin B12 and omega-3 fatty acids (both at 18%). Similarly, a recent survey of physicians in SA found that 75.7% of responders used melatonin as a sleep aid (Alateeq et al. Deemah et al., 2021). Another cross-sectional study in SA reported that 32.1% and 25.9% of the general population used vitamins/multivitamins and fatty acids, respectively (Algaeed et al., 2019). Previous reports have shown that multivitamins, omega-3 fatty acids, and vitamin D are the most commonly used DSs (Algaeed et al., 2019, Axon et al., 2017, Kennedy, 2005; Mishra Suruchi et al., 2021). A national household survey in the U.S., which included about 9,585 non-institutionalized civilians, reported garlic (1.8%), Ginkgo (1.5%), and St. John’s Wort (1.2%) to be the most commonly used herbal medications and DSs used by individuals with psychiatric problems (Niv et al., 2010). Similarly, low rates of St. John’s Wort and Gingko use were found in our study.

Evidence regarding the benefits of using vitamin D to improve mental health is contradictory. A few studies have shown that vitamin D has a beneficial effect on mental health (Jamilian et al., 2019, Spedding, 2014), whereas others have reported that it had no significant effect on mental health (Brzezinski et al., 2005, Gowda et al., 2015). With regard to melatonin, even though several *meta*-analyses have reported a moderate effect on decreasing time to sleep onset and increasing sleep duration and quality (Brzezinski et al., 2005, Ferracioli-Oda et al., 2013, Sarris, 2014), the American Academy of Sleep Medicine (AASM) has recommended against using nutritional substances, such as melatonin, to treat chronic insomnia (Brzezinski et al., 2005). There is growing evidence regarding the neuroprotective benefits of vitamin B12 and omega-3 fatty acids in terms of supporting cognitive function (Rathod et al., 2016). Vitamin B has also been shown to improve mood (Long and Benton, 2013).

Despite limited evidence regarding the efficacy and safety of DS use for mental health (Gracious et al., 2012, Sarris, 2014), many people still use them. Moreover, the tendency to use DSs was shown to increase with age (Mishra Suruchi et al., 2021, Niv et al., 2010). In our study, the age of responders using DSs was numerically higher than that of non-DS users (38.63 [14.38] vs. (36.18 [11.58)]) respectively. Consistent with previous reports, our study found that DS use was higher among females, although there was no significant difference in terms of sex between DS users and non-users (Kennedy, 2005; Mishra et al. Suruchi et al., 2021). Additionally, we observed an increase in DS use with higher education levels, similar to that reported by a previous national survey conducted in the U.S. (Niv et al., 2010).

Participants who were employed and those with a high monthly income (>15000 SAR) had a higher rate of DS use. This finding contradicts the common perception that patients with lower socioeconomic status tend to use DS because prescribed medications are expensive, especially since only 0.7% of DS users reported believing that psychotropic/sleep medications are expensive. Consistently, a survey of pharmacy students in the U.S. reported that none of the respondents admitted that the reason for using DS was the high cost of prescription or over-the-counter medications (Axon et al., 2017). A national survey in the U.S. showed that herbal medication and DS use for mental illness were significantly more frequent among educated and employed subjects, as we saw in our study (Niv et al., 2010).

In this study, responders with a history of mental disorder diagnosis were almost three times more likely to use DS. These higher odds for using DSs may be attributed to the belief that DSs will improve their mental health or to following the advice of health professionals, as this was commonly reported here. In addition, the use of DSs may indicate that patients are unsatisfied with pharmacological treatment improvements and thus seek alternative options. In contrast, a previous report found no significant difference in the rate of psychiatric disorders between patients who used herbal supplements for mental illness and those who did not (Niv et al., 2010). Using a validated assessment tool, the ISI, to assess sleep disorders, we observed that responders with high ISI scores and sub-thresholds or moderate insomnia were more likely to use DSs. Moreover, melatonin, which may play a role in improving insomnia, was one of the most frequently used DS in our sample (Ferracioli-Oda et al., 2013).

It is noteworthy that most earlier surveys assessed DS use for maintaining and/or improving health, not exclusively for improving mental health, as in our study (Algaeed et al., 2019, Axon et al., 2017, Kennedy, 2005; Mishra Suruchi et al., 2021). We believe that this survey is one of the first few recent studies to assess the use of DSs for mental health problems. Furthermore, this study used validated translated tools to evaluate the responders’ depression, insomnia, and anxiety. However, this study does have some limitations. First, the cross-sectional survey design can entail some recall, self-report, or selection bias and cannot confirm causal relationships. Second, we used convenience sampling to distribute the survey electronically through social media, which may have negatively affected the credibility of the study findings. Third, most surveyors were female (73%), which may have skewed generalizability concerning the Saudi population.

## 5 Conclusion

The prevalence of DS use for mental health problems in SA is high (44 %). Responders who reported a history of psychiatric disorders or current insomnia had a higher chance of using DSs. This may indicate that patients with an increased need for treatment for such conditions are more likely to seek alternative non-pharmacological options and refer even to unreliable sources of information about these options. Therefore, increasing public awareness regarding the benefits and risks of these alternatives is essential. In addition, psychiatrists and healthcare providers must provide evidence-based information about DS for mental health improvement, refer patients to or create legitimate web-based sources that provide accurate and reliable information, and encourage the public to consult healthcare professionals before deciding to self-treat mental health problems.

## Ethical Approval

Ethical approval was obtained from the Institutional Review Board of Princess Nourah bint Abdulrahman University (PNU) and the Ethical Approval Committee (IRB Log number 21-0305), in accordance with the Declaration of Helsinki.

## Funding

This research was funded by 10.13039/501100004242Princess Nourah Bint Abdulrahman University Researchers Supporting Project number (PNURSP2022R2018), 10.13039/501100004242Princess Nourah Bint Abdulrahman University, Riyadh, Saudi Arabia.

## Declaration of Competing Interest

The authors declare that they have no known competing financial interests or personal relationships that could have appeared to influence the work reported in this paper.
