# Supplementary material for: The use of dietary supplements for mental health among the Saudi population: A cross-sectional survey
Source: Saudi Pharm J. 2022 Mar 29;30(6):742–9. doi: 10.1016/j.jsps.2022.03.017 (PMC9257885; doi:10.1016/j.jsps.2022.03.017)
Supplement: Supplementary data 1 [file mmc1.docx]

**Table S1. Source of information about DSs (N=443)**

| ***Source of information about DS*** | **N, (%)** |
| --- | --- |
| Internet | 214 (48.3) |
| Healthcare specialist | 168 (37.9) |
| Personal reading | 138 (31.2) |
| Medical resources | 98 (22.1) |
| Pharmacist | 87 (19.6) |
| Nutritionist | 63 (14.2) |
| Did not refer to any source | 56 (12.6) |
| Friends | 50 (11.3) |
| Family | 46 (10.4) |
| Psychiatrist | 42 (9.5) |
| Television | 6 (1.4) |
| Other resources | 4 (0.9) |
